# Divergence and Conservative Evolution of XTNX Genes in Land Plants

**DOI:** 10.3389/fpls.2017.01844

**Published:** 2017-10-26

**Authors:** Yan-Mei Zhang, Jia-Yu Xue, Li-Wei Liu, Xiao-Qin Sun, Guang-Can Zhou, Min Chen, Zhu-Qing Shao, Yue-Yu Hang

**Affiliations:** ^1^Institute of Botany, Jiangsu Province and Chinese Academy of Sciences, Nanjing, China; ^2^State Key Laboratory of Pharmaceutical Biotechnology, School of Life Sciences, Nanjing University, Nanjing, China

**Keywords:** land plants, XTNX genes, plant disease resistance genes, evolution, function divergence

## Abstract

The Toll-interleukin-1 receptor (TIR) and Nucleotide-binding site (NBS) domains are two major components of the TIR-NBS-leucine-rich repeat family plant disease resistance genes. Extensive functional and evolutionary studies have been performed on these genes; however, the characterization of a small group of genes that are composed of atypical TIR and NBS domains, namely XTNX genes, is limited. The present study investigated this specific gene family by conducting genome-wide analyses of 59 green plant genomes. A total of 143 XTNX genes were identified in 51 of the 52 land plant genomes, whereas no XTNX gene was detected in any green algae genomes, which indicated that XTNX genes originated upon emergence of land plants. Phylogenetic analysis revealed that the ancestral XTNX gene underwent two rounds of ancient duplications in land plants, which resulted in the formation of clades I/II and clades IIa/IIb successively. Although clades I and IIb have evolved conservatively in angiosperms, the motif composition difference and sequence divergence at the amino acid level suggest that functional divergence may have occurred since the separation of the two clades. In contrast, several features of the clade IIa genes, including the absence in the majority of dicots, the long branches in the tree, the frequent loss of ancestral motifs, and the loss of expression in all detected tissues of *Zea mays*, all suggest that the genes in this lineage might have undergone pseudogenization. This study highlights that XTNX genes are a gene family originated anciently in land plants and underwent specific conservative pattern in evolution.

## Introduction

Plants have evolved two layers of immunity system against environmental pathogens (Dangl et al., 2013). The first layer detects infectious microbes by recognizing conserved pathogen-associated molecular patterns (PAMPs), including bacterial flagellin and fungal polysaccharides through plant receptor-like proteins that are located on the cell surface, and is therefore called PAMP-triggered immunity (PTI). Upon successful delivery of virulence factors (also termed effector proteins) by pathogens into plant cells to block the activation of PTI, the second line of plant defense are activated by directly recognizing the effector proteins or their modulating on host proteins to initiate effector triggered immunity (ETI). Activation of plant ETI largely depends on intracellular proteins that are encoded by plant disease resistance (R) genes (DeYoung and Innes, 2006). To date, over 100 functional R genes have been identified, of which >80% belong to the nucleotide-binding sequence and leucine-rich repeat (NBS-LRR) gene family.

Proteins encoded by intact NBS-LRR genes usually possess a conserved NBS domain, which is accompanied by a highly variable C-terminal LRR domain and a subclass-specific N-terminal domain. We previously reported that NBS-LRR genes have diverged into three subclasses prior to the radiation of angiosperms (Shao et al., 2016b). Based on the presence of a Toll/interleukin-1 receptor (TIR), coiled coil (CC), or Resistance to Powdery mildew8 (RPW8) domain at the N-terminal of the translated protein, the three NBS-LRR subclasses are designated as TIR-NBS-LRR (TNL), CC-NBS-LRR (CNL), and RPW8-NBS-LRR (RNL) genes, respectively (Shao et al., 2016b). Phylogenetic analysis has demonstrated that rounds of gene expansion have occurred in both TNL and CNL genes of angiosperms in the past 100 million years, although TNL genes have been completely lost in the monocot lineage and several dicot species (Collier et al., 2011; Shao et al., 2016b).

In addition to the structurally intact NBS-LRR genes encoding all three characteristic domains, massively truncated NBS-LRR genes were generated due to partial duplication of intact NBS-LRR genes (Shao et al., 2014; Zhang et al., 2016). In addition to these truncated genes with a characterized NBS domain, a collection of non-NBS genes encoding the TIR domain was also identified in land plants (Meyers et al., 2002; Sun et al., 2014). Several truncated proteins also play important roles in plant disease resistance. For example, the RPW8 genes in Brassicaceae that exhibit broad spectrum resistance against powdery mildew in *Arabidopsis* are truncated versions of the RNL genes (Xiao et al., 2001; Collier et al., 2011). Furthermore, several recent studies have revealed that some TX or TN genes are involved in plant disease resistance through different mechanisms (Kato et al., 2014; Zhao et al., 2015; Liu et al., 2017; Nishimura et al., 2017). For example, the truncated NBS-LRR protein TIR-NBS2 is required for exo70B1-mediated immunity by interacting and stabilizing the kinase activity of a calcium-dependent protein kinase (Liu et al., 2017). In contrast, the TIR-only protein RBA1 activates cell death in *Arabidopsis* by direct recognizing a pathogen effector (Nishimura et al., 2017).

An interesting question was then raised regarding whether these truncated genes were all derived from NBS-LRR genes via partial gene duplication or have a different origin. Phylogenetic analysis of the TNL, TN, and TX genes in the *Arabidopsis thaliana* genome revealed that TX and TN families were derived from and have co-evolved with the TNL families (Meyers et al., 2002). However, a comprehensive survey of TIR domain encoding genes in green plants revealed that a group of genes containing only the TIR domain (termed the T gene) exists in green algae (Sun et al., 2014). Because no TNL genes have been found outside the land plants to date, this finding suggests that plant genes encoding the TIR domain may have a heterogeneous origin.

The XTNX gene family is a small group of genes that was initially detected in *Arabidopsis* and rice by two pioneer studies on plant disease resistance genes (Bai et al., 2002; Meyers et al., 2002). Although TIR and NBS domains have been identified in each of their translated protein sequences, these have not been assigned as TNL genes unambiguously. This is largely due to the fact that both domains in the XTNX genes are too divergent to be recognized as typical TIR or NBS domains of NBS-LRR genes. Furthermore, unlike the TNL genes that have been completely lost in the monocot and several dicot lineages, XTNX genes are present in both monocots and dicots. In addition, phylogenetic analysis indicates that XTNX genes belong to a distinct clade from that of TX and TNL genes (Nandety et al., 2013), thereby suggesting that XTNX genes were not directly derived from the TNL genes. Interestingly, functional study revealed that *Arabidopsis* XTNX genes also have functions related to plant defense (Nandety et al., 2013). Overexpression of an XTNX gene significantly enhanced *A. thaliana* resistance to two different pathogens (Nandety et al., 2013), suggesting XTNX genes may represent a novel class of conservatively evolved plant disease resistance genes.

In the present study, to obtain further insights into the origin, evolution, and functional divergence (FD) of the XTNX genes, we comprehensively identified and conducted a phylogenetic reconstruction of homologs in 59 plant genomes of different lineages of green plants. Our findings demonstrated that the XTNX genes originated from a common ancestor of all land plants. In contrast to the extensive expansion of TNL genes or moderate expansion of T genes, only a single copy gene of each early diverged XTNX lineage was retained in most plant genomes. Expression analysis suggested that XTNX genes potentially play important roles in plant biological processes and functional differentiation may have occurred among different lineages.

## Results

### Identification of XTNX-Encoding Genes in 59 Plant Species

**FIGURE 1:**
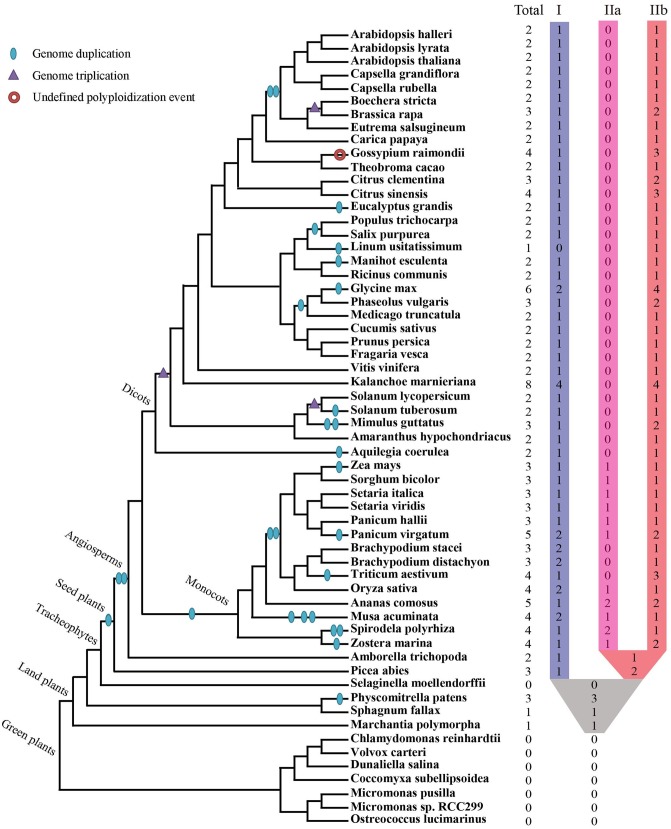
Identification and distribution of the XTNX genes across green plants. The phylogenetic relationship and WGD/WGT events were inferred from (Ruhfel et al., 2014; Panchy et al., 2016; Feng et al., 2017; Van de Peer et al., 2017; Xu et al., 2017). The total number of XTNX genes and their classifications in each species are shown.

Collectively 59 species, including green algae, mosses, lycophytes, one gymnosperm, as well as diverse families of angiosperms, were selected to represent major evolutionary nodes in the plant kingdom (**Supplementary Table S1**). A total of 143 XTNX-encoding genes were identified from the genomes of the 59 plant species (**Supplementary Table S2**), but no gene was identified in seven algae genomes (Chlorophyta) or the lycophyte *Selaginella moellendorffii*. The absence of the XTNX genes in the algal species suggests that the XTNX genes in the green lineage might have originated from the common ancestor of land plants, while *Selaginella* may have lost the XTNX genes due to its sharply reduced genome. XTNX genes were detected at lower copy numbers from the rest 51 land plant genomes (except for *S. moellendorffii*), varying from only 1 copy to the maximum of 8 copies (**Figure 1** and detailed in **Supplementary Table S2**). Of all species surveyed, *Kalanchoe marnieriana* possessed the highest number of XTNX genes (8 copies), whereas only one XTNX gene was observed in *Marchantia polymorpha*, *Sphagnum fallax*, and *Linum usitatissimum*. In addition, two XTNX genes were identified in 23 species, three genes in fourteen species, four genes in seven species, five genes in two species (*Ananas comosus* and *Panicum virgatum*), and six genes in one species (*Glycine max*).

Gene structure analysis revealed that most XTNX genes are intronless. Only 23 XTNX genes from 143 have annotated introns (**Supplementary Table S3**). The intron number in these genes varies from 1 to 8 (**Supplementary Table S3**). Amino acid sequence analysis revealed that although the TIR domain could be detected directly in nearly all XTNX genes using the online NCBI Conserved Domain Database, the NBS domain in XTNX proteins is too divergent to be identified for many sequences (**Supplementary Table S4**). Furthermore, the NBS domain in XTNX proteins is only half the length of a regular NBS domain of NBS-LRR genes, and is often annotated as AAA or P-loop superfamily domains by the online NCBI Conserved Domain database (**Supplementary Table S4**).

### Phylogenetic Analysis of XTNX Genes in the Plant Kingdom

**FIGURE 2:**
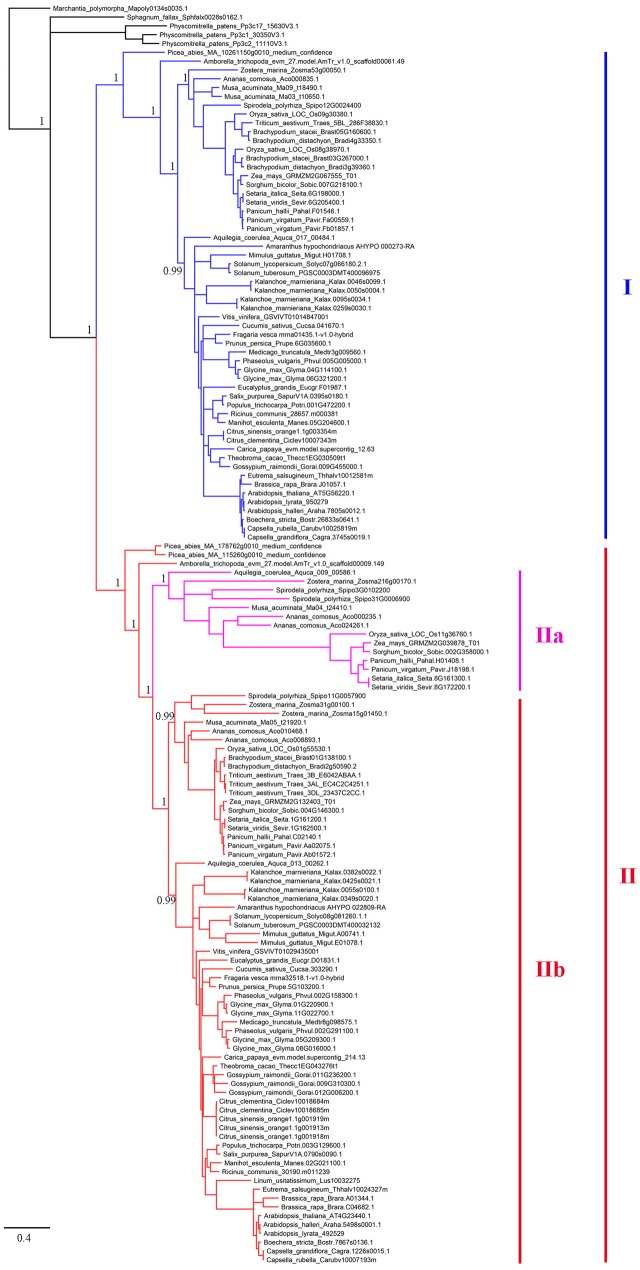
Phylogenetic analysis of the XTNX genes from 51 land plants. An ML tree was constructed by taking the alignment of all coding sequences as input. The support values (SH-aLRT value) for basal nodes are indicated. Genes belonging to clades I, IIa, and IIb are shown in different colors.

To establish the evolutionary history of XTNX genes in land plants, a phylogenetic tree was constructed. The presence of one XTNX gene in two species, *M. polymorpha* and *S. fallax*, from the basal land plant lineages suggests that the common ancestor of land plants possessed only one XTNX gene. Therefore, the *M. polymorpha* gene was used to root the XTNX gene tree. Assessment of the tree topology suggests that two rounds of ancient gene duplication occurred during the evolution of the XTNX genes (**Figure 2**). The earlier one occurred in the common ancestor of seed plants and resulted in the formation of two XTNX clades (clades I and II), while the later one took place in the common ancestor of monocots and dicots, causing the further divergence of clade II (subclades IIa and IIb). The genes in subclade IIa were only detected in some monocot species and one basal dicot species (*Aquilegia coerulea*), suggesting that XTNX genes of subclade IIa were lost in dicot ancestors soon after the split of *Aquilegia* lineage. In addition, the branch length of subclade IIa was longer than the other clades, which indicates that this lineage evolved rapidly. Besides the two ancient duplication events, some lineage- and species-specific duplications could also be detected, which further shaped the XTNX diversity in lateral diverged species.

### Motif Differences and Function Divergence among Various XTNX Lineages

**FIGURE 3:**
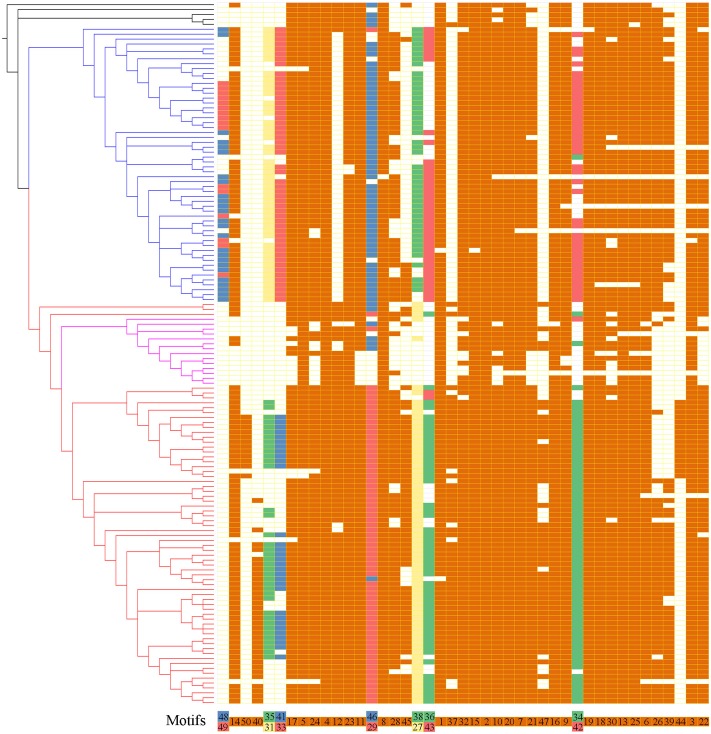
The motif composition of the XTNX genes on the background of their phylogenetic relationship. The phylogenetic relationship of all XTNX genes are displayed as determined in **Figure 2**. A total of 50 motifs were identified from 143 full-length XTNX amino acid sequences. For each gene, the identified motifs were arranged according to the order they occur in the amino acid sequence, as indicated in the bottom of the panel. A colored box indicates presence of a motif, whereas a white box indicates its absence. Detailed information on the 50 motifs is provided in the **Supplementary Table S5**.

To further reveal the diversification of XTNX clades/subclades, we analyzed their conserved motifs using the MEME motif detection software. The motif compositions were listed and used to compare different clades (**Supplementary Table S5**). Among the 50 identified motifs, motifs 1 and 3 have the maximum lengths of 50 amino acids, whereas motifs 39 and 47 showed minimum motif lengths of 10 amino acids. The average motif length of 50 conserved motifs was 21.9. **Figure 3** shows that motifs 2 and 4 occur in all species. Similarly, more than 20 motifs (motifs 1, 3, 5, 6, 7, 8, 9, 10, 11, 13, 14, 15, 16, 17, 18, 19, 20, 21, 22, 23, 24, 25, 30, and 32) appeared in most of the identified XTNX genes (>80%). There were also several clade-specific motifs; for example, motifs 31, 33, 38, 43, 48, and 49 are present in clade I but not in clade II XTNX genes, whereas motifs 27, 35, 40, 41, 44, 47, and 50 only occur in clade II genes. This result suggesting sequence divergence has occurred after the separation of clade I and II. Motifs 35 and 41 were lost in subclade IIa, and only appeared in subclade IIb. Motifs 44 and 50 were Poaceae species-specific in subclade IIb, whereas motif 40 was dicot species-specific in subclade IIb. When putting the motif profile of the XTNX genes under the background of its phylogeny (**Figure 3**), several motif gain and loss events could be easily detected. For example, the subclade IIb monocot XTNX genes gained lineage-specific motifs 44 and 50, whereas dicot XTNX genes within this subclade obtained motif 40. In contrast, frequent motif loss was detected in subclade IIa genes, suggesting a pseudogenization or neofunctionalization process.

In addition to gain and loss of clade-specific motifs, amino acid substitution would also cause functional diversification (FD) of XTNX genes. To estimate FD among the three major clades of XTNX genes, the DIVERGE3.0 program (Gu et al., 2013) was adopted to analysis XTNX amino acid sequences with two different models. The type I FD was estimated based on evolutionary rate (Gu, 1999), whereas type II FD was estimated based on biochemical property difference of amino acids (Gu, 2006). The result revealed that significant type I FD could be detected between each tested pair of XTNX clades (**Table 1**). This is in accordance with the motif difference pattern and further supported FD has occurred after the separation of the three XTNX clades.

**Table 1:** Analysis of FD among different clades.

FD	Clades	Coefficient (𝜃) ± SE (P)
Type I	Clade I versus IIa	0.432472 ± 0.096382 (1.07E-08)
	Clade I versus IIb	0.563411 ± 0.086543 (0.00E+00)
	Clade IIa versus IIb	0.277236 ± 0.091151 (1.15E-04)
Type II	Clade I versus IIa	-0.070316 ± 0.285414
	Clade I versus IIb	-0.107782 ± 0.308432
	Clade IIa versus IIb	-0.28179 ± 0.373912

### Mechanisms for XTNX Gene Duplication and Contraction

As supported by the phylogenetic analysis, two ancient gene duplication events occurred during XTNX gene evolution in land plants. To explore whether these two rounds of duplication are consequences of ancient polyploidization, we performed within-genome collinearity analysis. The collinearity of the XTNX genes from subclades IIa and IIb, or subclade I/II was not detected in any surveyed species, suggesting that these did not result from ancient polyploidization. This result is in accordance with the current knowledge on plant genome polyploidization that no whole genome duplication (WGD) or whole genome triplication (WGT) event has been documented in the two divergent nodes of land plants.

**FIGURE 4:**
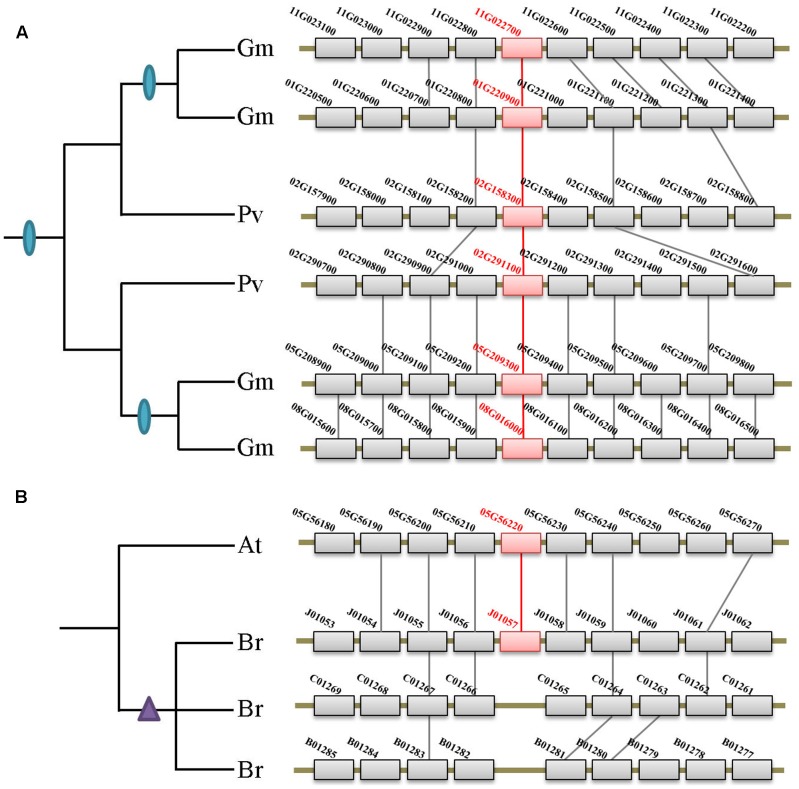
Syntenic analysis of the XTNX genes in Fabaceae and Brassicaceae species. **(A)** Two rounds of WGDs resulted in the XTNX genes in legume species. Collinearity between two *Phaseolus vulgaris* blocks containing two XTNX genes (02G158300 and 02G291100) resulted from the ancient WGD in the common ancestor of legume, whereas another collinearity block pair containing XTNX genes from soybean (chromosomes 01G versus 11G, and chromosomes 05G versus 08G) resulted from a soybean-specific WGD event. **(B)** Rapid loss of XTNX duplicons after the WGT in the *Brassica* *rapa* genome. Among the three chromosomal blocks that resulted from WGT in *B. rapa*, two of these have lost the XTNX genes within the blocks.

Besides the two ancient duplication events, there were also many lineage/species-specific duplications as suggested by more than one gene in a genome clustered in the same clade/subclade on the tree (**Figure 2**). To examine whether some of these duplicons are resulted from lineage-specific WGDs/WGTs, we performed collinearity analysis for all genes within each genome that fall into the same lineage. Our data revealed that 19 of the 28 lineage/species-specific duplicated genes are present on the syntenic blocks, and 10 of them are resulted from recently occurred WGDs/WGTs. For example, four soybean genes in subclade IIb are resulted from two rounds of WGDs that occurred 54 million years ago in the common ancestor of legumes and 10 million years ago in soybean, respectively (**Figure 4A**). Apart from WGD/WGT event that resulted in gene duplication, examples of tandem duplication were detected, e.g., the three *Citrus sinensis* genes (orange1.1g001913m, orange1.1g001918m, and orange1.1g001919m) and two *C. clementina* genes (Ciclev10018684 and Ciclev10018685) in subclades IIb. There were also some duplications that could not be determined as segmental or tandem duplications, which were designated as ectopic or other duplications (**Supplementary Table S2**).

According to several recent studies, angiosperms have undergone rounds of ancient and lineage-specific WGDs/WGTs. However, our data revealed that gene duplication across different XTNX lineages was observed only in certain species, whereas most angiosperms retained only one XTNX gene in both subclades I and IIb. This suggests that in most species, XTNX genes have undergone frequent gene loss following WGDs/WGTs. **Figure 4B** shows such an example in Brassicaceae, wherein *Brassica rapa* underwent genome triplication after its separation from the Arabidopsis lineage, yet only retained a single XTNX gene in clade I, with two of the three syntenic blocks have lost their XTNX genes soon after the WGT event.

### Expression Pattern of the XTNX Genes

**FIGURE 5:**
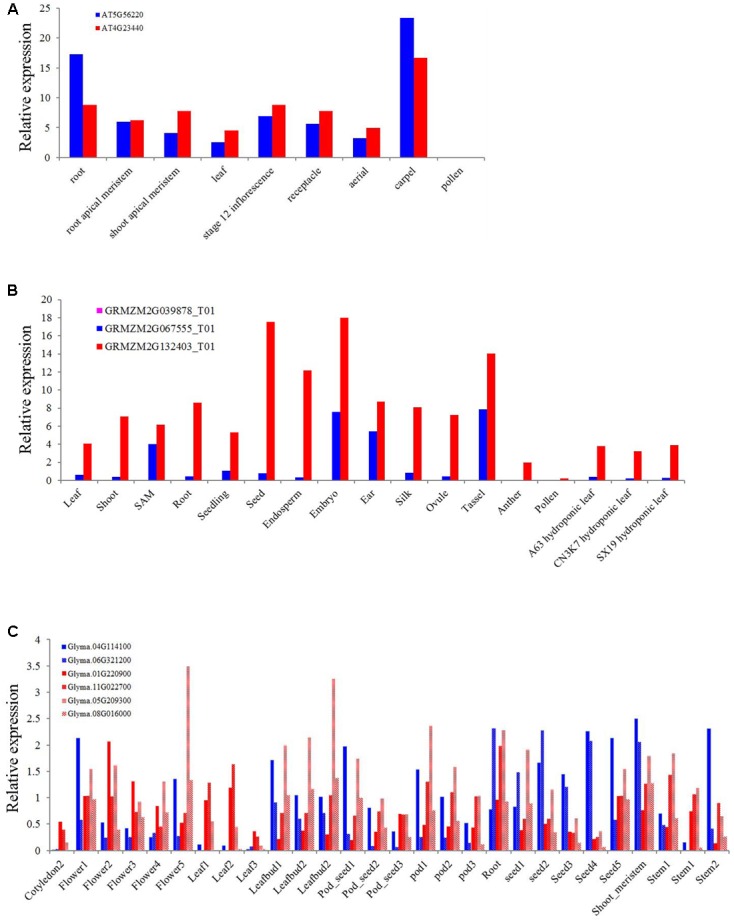
Expression of XTNX genes in different tissues of three angiosperms. The relative expression of XTNX genes in **(A)** *Arabidopsis thaliana*, **(B)** *Zea mays*, and **(C)** soybean.

As one of the most conserved TIR or NBS domain containing genes, the exact function of the XTNX genes has not been well determined. To obtain some clues for the biological function of XTNX genes, we determined the expression of genes from different XTNX lineages in three angiosperm genomes, including *A. thaliana*, soybean, and *Zea mays* from several public data sets. **Figure 5A** shows that the two *Arabidopsis* XTNX genes At5g56220 (clade I) and At4g23440 (clade IIb) are expressed in all tissues except for pollen. In most tissues, At4g23440 has relatively higher expression than At5g56220, although whether the differential expression between the two genes is significant could not be detected in the used data sets. The *Z. mays* genome contains three XTNX genes named GRMZM2G067555 (clade I), GRMZM2G039878 (clade IIa), and GRMZM2G132403 (clade IIb). While the clade I and clade IIb XTNX genes are also differentially expressed in most tissues as observed in *Arabidopsis*, the expression of the clade IIa gene GRMZM2G039878 was not detected in any tissue (**Figure 5B**), which suggest that genes in this clade may either only express under certain stimulations or have underwent pseudogenization rather than neofunctionalization process.

Because genome duplication events have resulted in gene duplication of the two retained XTNX subclades in soybean, we then explored whether these recent duplicated genes maintained similar expression pattern. **Figure 5C** shows that these recent duplicated gene pairs (Glyma.04G114100 vs. Glyma.06G321200, Glyma.01G220900 vs. Glyma.11G022700, and Glyma.05G209300 vs. Glyma.08G016000) that derived from the ∼10 million-year Glycine WGD also show different expression patterns. For example, the expression of Glyma.04G114100 in one sample (stem 2) is almost five times of Glyma.06G321200. This suggested that at least in some species, recent duplicated XTNX genes became to express differentially. However, more detailed experiments should be devoted to test expression difference between specific gene pairs.

## Discussion

### XTNX Genes Are Different from NBS-LRR Genes at Several Aspects

Plants have a great number of NBS or TIR domain-encoding genes, which are represented by the largest plant disease resistance gene family NBS-LRR genes and massive TN, TX, and T genes (Sun et al., 2014; Shao et al., 2016b). While the origin and evolution of these genes in plants have been extensively studied (Xue et al., 2012; Sun et al., 2014), the evolutionary history of XTNX genes, a gene family composed of degenerate TIR and NBS domains, has yet been well elucidated. The XTNX genes were first reported in *Arabidopsis* and rice genomes by two pioneer studies on plant disease resistance genes (Bai et al., 2002; Meyers et al., 2002). Although an early study proposed that TX and TN families were derived from and have co-evolved with the TNL families, recent phylogenetic analysis revealed that XTNX genes are clustered into a single clade that is closely related to while distinct from the TX and TNL genes (Meyers et al., 2002; Nandety et al., 2013), suggesting an independent origin of the XTNX genes. Several features of gene and protein structure provide additional evidence that supports the idea that the XTNX genes are different from the NBS-LRR genes. First, similar to the two XTNX genes in *Arabidopsis*, nearly all XTNX genes found in land plants are intronless (**Supplementary Table S3**). For those XTNX genes that do have annotated introns, none of these were conserved among the XTNX genes of different plants. In contrast, all the three subclasses NBS-LRR genes have conserved introns (Xue et al., 2012; Shao et al., 2016b). For example, the TNL genes of both mosses and angiosperms have at least two conserved introns with characterized intron phase of 2 and 0, respectively. The first intron presented between TIR and NBS domains is also conserved in truncated TN genes that were derived from TNL genes. Second, the degenerate NBS domain that is highly conserved among XTNX genes in all land plants is only half the length of the NBS domain of the NBS-LRR genes. Among several conserved motifs (including P-loop, Kinase 2, RNBS-B, GLPL, and RNBS-D) in the NBS domain of NBS-LRR genes (Shao et al., 2016b), only one of them (the P-loop motif) could be detected in XTNX genes. Furthermore, the identification of an XTNX gene in *M. polymorpha* indicated that XTNX genes are at least as old as the NBS-LRR genes, which also rejects the theory that it is a recently diverged lineage from TNL or NBS-LRR genes in land plants. Taken together, both phylogenetic analysis and gene structure characteristics support the idea that XTNX genes have separated with NBS-LRR genes prior to the radiation of land plants if they do have a common ancestor.

### Ancient Divergence and Contractive Evolution of the XTNX Genes

The TIR domain occurs widely in the three life domains, which thereby are involved in different structures by fusing with other domains. In plants, several TIR only genes were detected, and their origin could be traced to green algae (Sun et al., 2014). The largest TIR domain-containing gene family in plants is the TNL subfamily of NBS-LRR family genes, which has only been detected in land plants to date (Xue et al., 2012; Sun et al., 2014). In the present study, we detected the XTNX genes in nearly all land plant genomes, but not in seven green algal species, thereby suggesting that the XTNX genes may have originated early soon after plants colonized the terrestrial environment. The XTNX genes underwent two rounds of ancient duplications during land plant evolution, one caused the separation of clades I and II, and another the clade II-specific duplication took place prior to the divergence of dicots and monocots, thereby resulting in the formation of clade IIa and IIb. Although a polyploidization event has been documented in the creation of new NBS-LRR subclades, such as the two RNL lineages (Shao et al., 2016b), we found no evidence supporting the two XTNX duplications was also created by ancient polyploidization events. However, we should cautious that severe genomic fractionation after WGD/WGT may greatly reduce the identification of ancient polyploidization event.

Besides several documented ancient polyploidization events in the common ancestor, different angiosperms have also experienced intensive lineage-specific polyploidization events, especially during the Cretaceous-Paleogene (K-P) boundary (Vanneste et al., 2014). Theoretically, a polyploidization event could cause gene duplication at whole genome level. In a few angiosperms, such as the common bean and soybean, recent WGDs/WGTs indeed caused XTNX gene duplications. Among the 28 duplications found in the XTNX genes, 10 of them were caused by WGDs/WGTs, whereas only 3 of these were identified as tandem duplications. This is in contrast to NBS-LRR genes, for which most duplications occurred in tandem (Shao et al., 2014).

As for the extent of gene duplication, unlike the great expansion of NBS-LRR genes in angiosperm families (Shao et al., 2016b), most species only retained one copy of XTNX genes in clades I and IIb, with some monocots and one dicot genome also retaining one gene in clade IIa (**Figure 2**). Considering that several independent WGD/WGT events occurred in different angiosperm lineages during the K-P boundary (Vanneste et al., 2014), our data suggest that the XTNX genes in almost all surveyed species lost their duplicated copies invariably soon after the independent WGD/WGT events and coincidently evolved in a contractive manner.

### Different Evolutionary Fates and Functional Divergence of XTNX Lineages

Three groups of early diverged XTNX genes exhibit distinct evolutionary patterns as observed in our phylogenetic analysis. Two groups of these genes evolved conservatively in both dicots and monocots, with most species containing only one copy of the gene in each lineage. This evolutionary pattern differs from that of the TNL genes, which have experienced furious expansion since the origin of angiosperm (Shao et al., 2016b). Even the most conserved NBS-LRR subfamily, RNL, shows greater copy number variation in its two angiosperm lineages than the two XTNX lineages (Shao et al., 2016b). The conservative evolution of XTNX genes suggests that both lineages may have developed important and conserved biological functions (De Smet et al., 2013). Motif analysis revealed that independent sequence innovations by acquisition of novel lineage-specific motifs occurred after the two lineages diverged (**Figure 3**), indicating functional differentiation of the two lineages. This difference was also supported by our expression analysis of genes of the two different lineages in three different species. In both *Arabidopsis* and *Z. mays*, the clade IIb lineage genes showed higher expression than clade I genes in most of the tissues assessed. Comparing with the conservative evolution of clades I and IIb, clade IIa genes are more likely to have undergone pseudogenization, as indicated by the long branch on the tree, the frequent loss of ancestral motifs, and the undetectable expression levels in all tissues. However, we could not rule out the possibility that genes in this clade might have undergone neofunctionalization and are expressed specifically in certain organs or life stages which have not been detected by current experiments.

Nearly all TIR or NBS domain containing genes in plants with known functions are involved in plant disease resistance (Liu et al., 2007). The TIR domain is also presented in animal immune receptor genes (Casanova et al., 2011). Although the exact function of XTNX genes is unknown, a recent study showed that the overexpression of an *Arabidopsis* XTNX gene enhances its resistance to the bacterial pathogen *Pseudomonas syringae* pv. tomato DC3000 and a necrotrophic fungal pathogen *Fusarium oxysporum* (Nandety et al., 2013). However, unlike most R genes that show tissue-specific or pathogen-induced expression, both XTNX genes from clades I and IIb are expressed in nearly all tissues except pollen. Furthermore, plant R genes are usually under strong selection pressure from rapidly changed pathogen environment and exhibit frequent “birth to death” evolutionary pattern (Shao et al., 2016a). No R gene has been reported to show a conservative evolutionary pattern across land plants like that of the XTNX genes. Therefore, we speculate that XTNX genes are not pathogen detectors, as observed for most NBS-LRR genes. Recent studies have revealed that there are some NBS-LRR genes involved in plant disease resistance that function as signal transductors (e.g., RNL genes and the TN2 gene) (Collier et al., 2011; Liu et al., 2017). Given the expression patterns observed and its conservative evolutionary pattern, we favor the notion that XTNX genes participate in plant disease resistance by signal transduction if they were indeed involved in plant disease resistance. Additionally, the constitutive expression of the two XTNX genes in most detected tissues from different plants suggests that these may have undetected functions in plant biological processes other than defense. Further experimental studies are still needed to explore the full function of these conserved TIR domain containing genes.

## Materials and Methods

### Data Used in This Study

A total of 59 genomes from different plant species were used, including seven species from Chlorophyta, three species from Bryophyta, one species from Lycophyta, one species from Gymnospermae and 47 species from Angiospermae. The genome sequence of *Picea abies* and its annotation files were obtained from ftp://plantgenie.org/Data/ConGenIE/Picea_abies/v1.0/FASTA/. The other 58 plant genome sequences and annotation files were downloaded from the Phytozome database^1^ (**Supplementary Table S1**).

### Identification of XTNX-Encoding Genes

To identify the XTNX genes in each genome, the amino acid sequences of two known XTNX genes (At4G23440 and At5G56220) in *A. thaliana* were used as queries to perform BLASTp searches, with the threshold expectation value set to 0.001. All hit sequences were then analyzed with Conserved Domains Database (CDD^2^) and then manually checked to confirm the presence of both TIR and NBS domains. When two or more transcripts were annotated for a gene, the most intact one was selected. The exon position and intron phase of each gene were transformed from the GFF3 file of the reference genome. Genes with very short TIR or NBS domains less than half of those in *A. thaliana* XTNX genes were eliminated from the data sets.

### Sequence Alignment and Phylogenetic Analysis

Sequence alignment and phylogenetic analysis were performed as described in our previous study (Shao et al., 2014). Briefly, the full-length coding sequences of XTNX genes were aligned using ClustalW with default options and then manually corrected in MEGA 7.0 (Kumar et al., 2016). Then, the alignment (**Supplementary Data Sheet 1**) was analyzed in MEGA 7.0 to select the optimal evolutionary model that adequately fit the sequence data. Finally, phylogenetic trees were generated with the maximum likelihood algorithm using PhyML software that integrated in the Seaview package (Gouy et al., 2010) with the GTR+T+I parameters. Branch support was assessed with the aLRT statistic (Guindon and Gascuel, 2003).

### Analysis of Conserved Motifs and Functional Divergence

The conserved motifs in the amino acid sequence of the XTNX genes were identified using the Multiple Expectation Maximization for Motif Elicitation 4.10.0 (Bailey and Elkan, 1995). The following parameters were used: the maximum number of motifs, 50; minimum motif and maximum widths, 10 and 50, respectively; and all other parameters were defaults. Individual profiles of each conserved motif were assessed, and only the conserved motifs with *P*-values ≤ 10^-4^ were reported. Motif composition for each XTNX protein was plotted against the phylogeny of XTNX genes. Clade-specific motifs were detected to demonstrate sequence divergence of different XTNX clades.

The DIVERGE version 3.0 software (Gu et al., 2013) was used to estimate FD among XTNX clades. Both the type I and type II FD were estimated based on either the occurrence of altered selective constraints or the radical shifts of physiochemical properties, respectively (Gu, 1999, 2006).

### Synteny Analyses

We used the online Plant Genome Duplication Database (PGDD^3^) to investigate the syntenic relationship of XTNX genes within and among species (Tang et al., 2008). For species that are not included in the PGDD, MCScan, a package used by the PGDD, was adopted to perform a synteny examination of paralogous genes among genomes through BLASTp searches.

### Expression Analysis of XTNX Genes

The expression data of *A. thaliana* XTNX genes (TPM) was retrieved from (Cheng et al., 2017). The expression data of *Z. mays* XTNX genes (FPKM) was obtained from (Thatcher et al., 2014). To analyze the expression of *Glycine max* XTNX genes, the raw transcriptome data of various tissues (Shen et al., 2014) were downloaded from GenBank, and the expression of each gene was calculated by mapping the raw reads onto the reference genome and normalized as FPKM.

## Author Contributions

Y-MZ, Z-QS, and Y-YH conceived and designed the project. Y-MZ and Z-QS obtained and analyzed the data. J-YX, L-WL, X-QS, G-CZ, and MC participated in the data analysis. Y-MZ drafted the manuscript. J-YX, Z-QS, and Y-YH modified the manuscript. All authors have read and approved the manuscript for publication.

## Conflict of Interest Statement

The authors declare that the research was conducted in the absence of any commercial or financial relationships that could be construed as a potential conflict of interest.

## Footnotes

1 http://www.phytozome.org/

2 http://www.ncbi.nlm.nih.gov/Structure/cdd/wrpsb.cgi

3 http://chibba.agtec.uga.edu/duplication/

## Supplementary Material

TABLE S1 A list of 59 genomes surveyed in this study.

TABLE S2 A list of 143 XTNX genes identified in 51 land plant genomes.

TABLE S3 Gene structure of 143 XTNX genes.

TABLE S4 TIR and NBS domains identified in XTNX genes.

TABLE S5 The 50 most significant motifs in the XTNX genes.

DATA SHEET 1 The alignment of XTNX genes.
