# Supplementary material for: Divergence and Conservative Evolution of XTNX Genes in Land Plants
Source: Front Plant Sci. 2017 Oct 26;8:1844. doi: 10.3389/fpls.2017.01844 (PMC5662649; doi:10.3389/fpls.2017.01844)
Supplement: TABLE S5 — The 50 most significant motifs in the XTNX genes. [file Table_5.DOCX]

| **Motif** | **Pictogram** | **Consensus** | **E-value** |
| --- | --- | --- | --- |
| 1 | 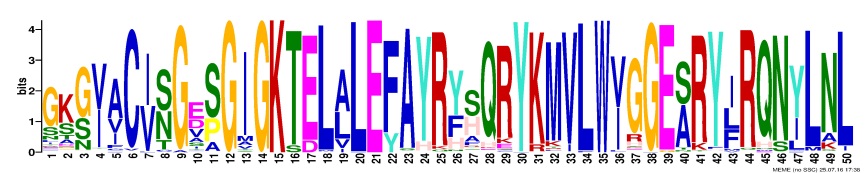 | GK[GS][VI]AC[IV][SN]GE[SP]GIGKTEL[AL]LEFAYR[YF][SH]QRYKMVLW[VI]GGE[SA]RY[ILF]RQN[YI]LNL | 2.7e-6211 |
| 2 | 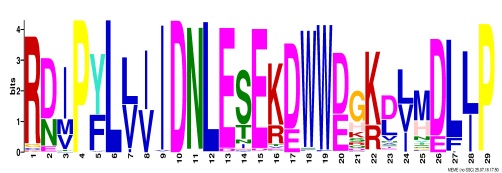 | R[DN]IP[YF]L[LV][IV]IDNLESE[KR][DE]WW[DE]G[KR][DL][LVI][MH]DL[LI]P | 1.5e-3438 |
| 3 | 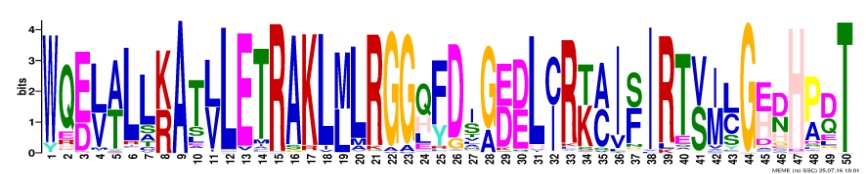 | WQ[ED][LV][AT]LL[KR]AT[LV]LETRAKL[ML]LRGG[QH][FY]D[IS][GA][ED][DE]L[CI]R[KT][AC]I[FS]IRT[VS][IM][LC]G[EH][DN]H[PA][DQ]T | 1.8e-5208 |
| 4 | 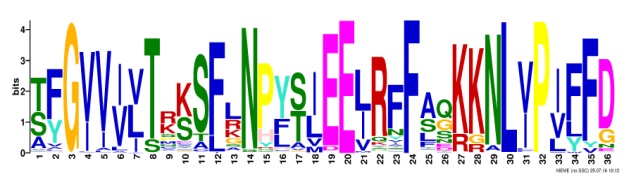 | [TS][FY]G[VI]V[IV][VL]T[RK][KS]SFLNP[YF][ST][IL]EE[LI]RFF[AS][QG]KKNL[VI]P[IV]FFD | 1.3e-3648 |
| 5 | 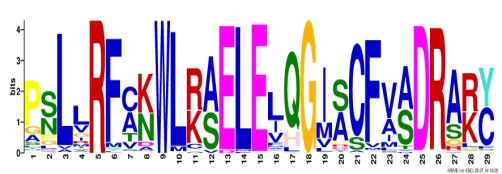 | P[SN]LLRF[CA][KN]WL[RK][AS]ELELQGI[SA]CF[VA][AS]DRA[RK][YC] | 8.6e-2872 |
| 6 | 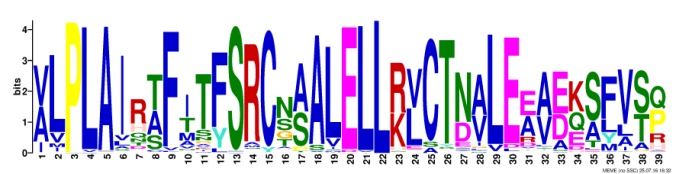 | [VA]LPLAIR[TA]F[IT]T[FY]SRC[NS][AS]ALELL[RK][VL]CT[ND][AV]LE[EA][AV][ED][KQ][SA]FV[ST][QP] | 1.4e-3541 |
| 7 | 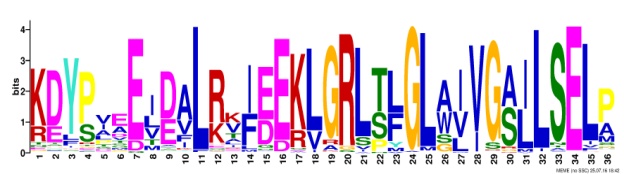 | K[DE]Y[PS]VEE[IL][DE][AV]L[RK][KV][IF][ED]EKLGRL[TS][LF]GL[AW][IV]VG[AS][IL]LSEL[PA] | 2.4e-3075 |
| 8 | 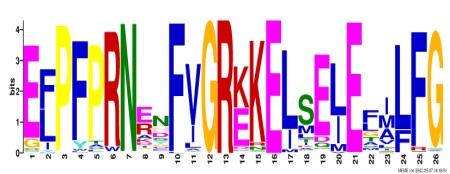 | E[FL]PFPRN[ER]NFVGR[KE]KELSE[LI]EF[IMA][LF]FG | 8.5e-2609 |
| 9 | 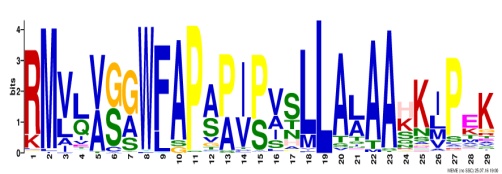 | RM[VL][LQ][VA][GS][GA]WFAP[AS][PA][IV][PS][VA]SLLA[LA]AA[HK]K[IL]PEK | 5.6e-2438 |
| 10 | 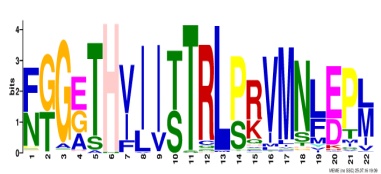 | [FN][GT]G[EGA]TH[VI][IL]I[TS]TRL[PS][RK]VMN[LF][ED]P[LM] | 6.6e-2108 |
| 11 | 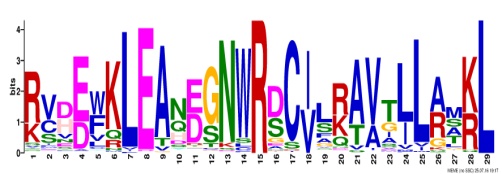 | [RK][VC][DH][ED][WF]KLEA[NQ][ED][GS]NWR[DS]C[IV]L[RK][AT][VA]T[LI]L[AR]M[KR]L | 6.4e-2390 |
| 12 | 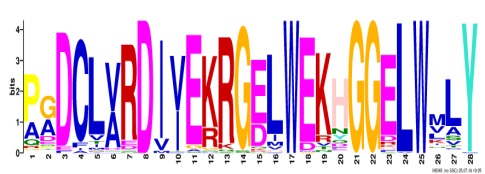 | [PA][GA]DCL[VA]RDI[VI]E[KR]RG[ED]LWEKHGGELWMLY | 3.1e-1795 |
| 13 | 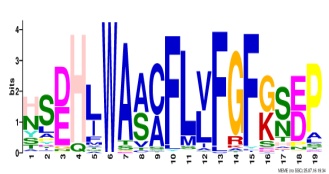 | [HN]S[DE]H[LI]WA[AS][CA]FL[VL]FGF[GK][SN][ED]P | 1.3e-1670 |
| 14 | 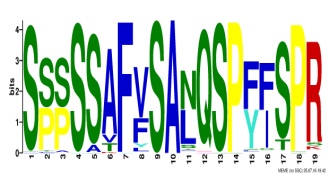 | S[SP][SP]SSAF[VF]SA[NL]QSP[FY][FI]SPR | 2.9e-1705 |
| 15 | 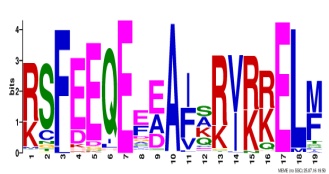 | [RK]SFEEQE[EF][EA]A[IF][SAK][RK][VI][RK][RK]EL[MF] | 2.2e-1636 |
| 16 | 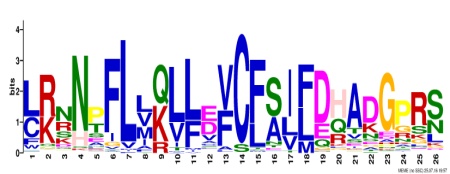 | [LC][RK][NR]N[PT]FL[LVM][QK][LV][LF][ED][VF]C[FL][SA][IL][FL]D[HQ]ADGPR[SN] | 2.5e-1766 |
| 17 | 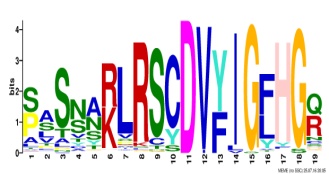 | [SP][AS]S[NS][AN][RK]LRSCDV[YF]IG[FL]HG[QR] | 2.1e-1535 |
| 18 | 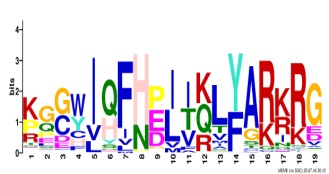 | [KP]G[GC][WY]I[QH]F[HN][PED][IL][ITV][KQR]L[YF]A[RK][KR]RG | 2.2e-1326 |
| 19 | 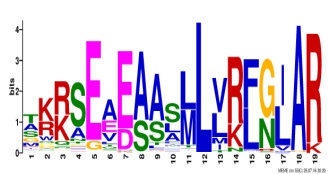 | T[KR][RK]SE[AE][ED][AS][AS]S[LM]L[VL][RK][FL][GN][IL]AR | 2.9e-1195 |
| 20 | 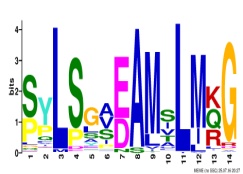 | [SP]YLSG[AVS][ED]AMSLM[KQR]G | 4.6e-947 |
| 21 | 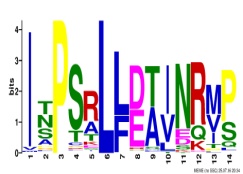 | I[TNS]PS[RA]L[LF][DE][TA][IV]NR[MV][PS] | 1.8e-917 |
| 22 | 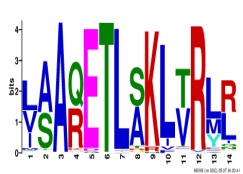 | [LVI][AS]A[QR]ETL[SA]KL[TV]RL[RL] | 1.3e-929 |
| 23 | 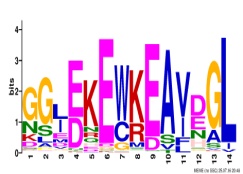 | GG[LI][ED]KE[WC][KR]EA[VI][DE]GL | 3.3e-901 |
| 24 | 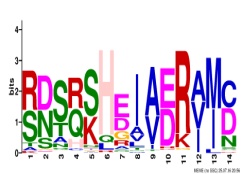 | [RS][DN]S[RQ][SK]H[ED]I[AV][ED]R[AVI][MI][CD] | 2.6e-833 |
| 25 | 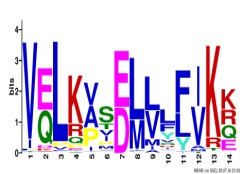 | V[EQ]LK[VAP]S[ED][LM][LV]L[FL][IV]K[KRQ] | 5.2e-776 |
| 26 | 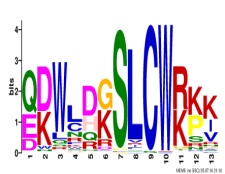 | [QE][DK]WL[DH][GK]SLCW[RK][KP][KI] | 7.0e-697 |
| 27 | 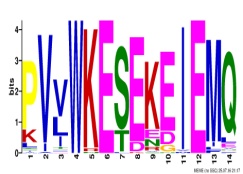 | PV[VL]WKE[ST]EKEIEMQ | 2.8e-673 |
| 28 | 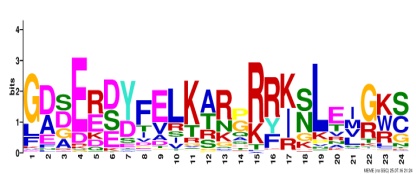 | G[DA][DS]E[RE][DSE][YD]FELK[AT]R[PG][RK][RY][KI][SN]LE[IM][GR][KWR][SC] | 2.9e-834 |
| 29 | 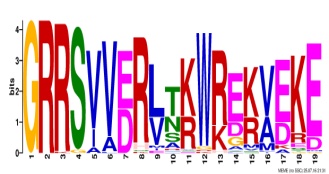 | GRRSVV[ED]R[LV][TN][KR]W[RK]E[KR][VA][ED]KE | 2.7e-756 |
| 30 | 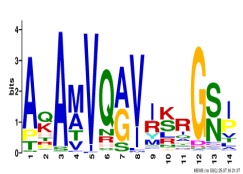 | A[QK]A[MA]VQ[AG][VI][IR][KS]RG[SN][IP] | 4.3e-655 |
| 31 | 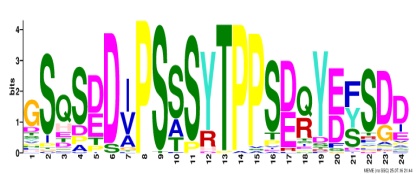 | GSQS[DE]D[IV]PS[SA]SYTPPS[DE][QR]Y[ED][FYS]S[DG]D | 5.6e-650 |
| 32 | 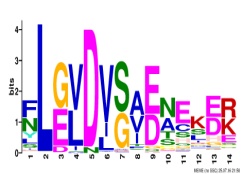 | [FN]L[GE][VL]DV[SG][AV][ED][NA]EK[ED][RK] | 3.7e-648 |
| 33 | 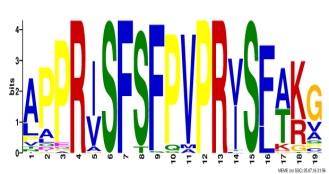 | APPR[IV]SFSFPVPR[VI]S[FL][AT][KR]G | 7.2e-633 |
| 34 | 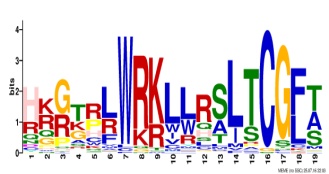 | H[KR][GR]T[RP][LR]WR[KR]L[LW]R[SA]LTCG[FL][TAS] | 5.4e-501 |
| 35 | 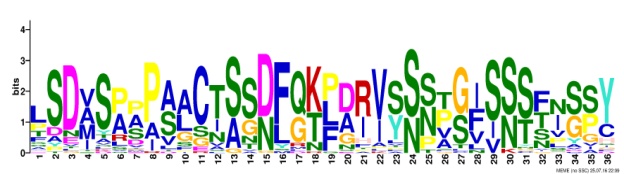 | LSD[VAM]S[PA][APS]P[AS][AL]CT[SA][SG][DN][FL][QG][KT][PFL][DA][RI][VI][SY][SN][SN][PT][GS][IF]S[SN][ST][FT][IN][SG][SP]Y | 9.3e-497 |
| 36 | 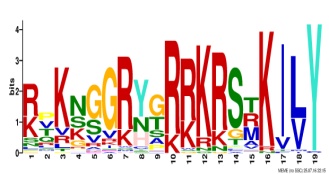 | [RK]PK[NKS][GS]GR[YN][GT]R[RK]KRS[TMR]KI[LV]Y | 8.0e-430 |
| 37 | 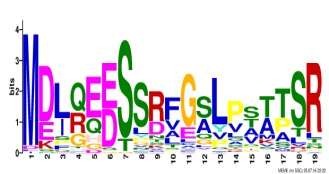 | M[DE][LI][QR][EQ][ED]SS[RD]FG[SA]LPS[TA][TP]SR | 1.9e-344 |
| 38 | 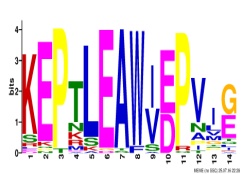 | KEPTLEAW[IV][ED]PVI[GE] | 6.9e-313 |
| 39 | 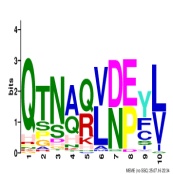 | QTN[AQ][QR][LV][DN][EP][YF][LV] | 6.4e-303 |
| 40 | 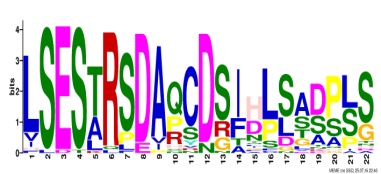 | LSES[TA]RSDA[QP]CDS[IF][HD][LP][SL][AS][DS][PS][LS][SG] | 1.6e-279 |
| 41 | 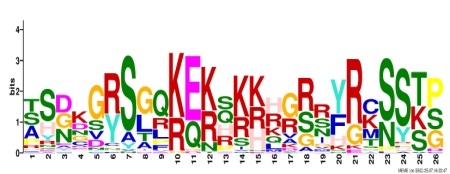 | [AST][SH][DGN]KG[RY]S[GL]Q[KR][EQ][KR][QSH][KR][KRH][HR][GR][RS][RS][YF][RG][CK][SN]S[TKS][PSG] | 1.4e-369 |
| 42 | 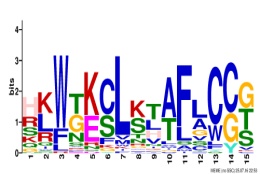 | H[KL]W[TG][KE][CS]L[KS][TL][AT]F[LA]C[CG][GTS] | 1.1e-169 |
| 43 | 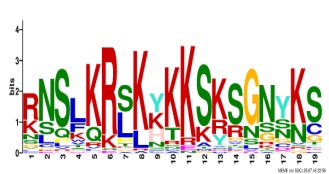 | [RK]NSLKR[SL]K[YKH]KKS[KR]SGNY[KN]S | 5.5e-210 |
| 44 | 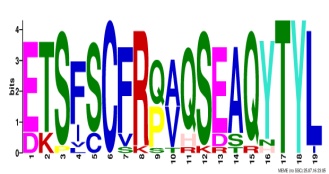 | ETSFSCFR[QP][AV]QSEAQYTYL | 3.2e-175 |
| 45 | 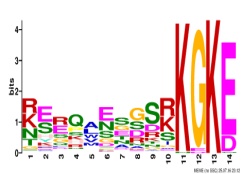 | [RK][ES][ER]QA[EN]SGS[RK]KGKE | 7.3e-164 |
| 46 | 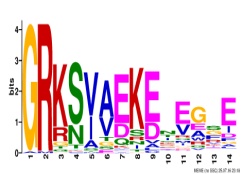 | GRK[SN][VI][AV]EK[ED]x[EV]GxE | 1.3e-183 |
| 47 | 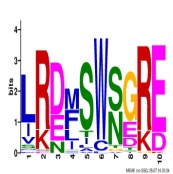 | LR[DE][MF]SW[SN]GRE | 5.2e-160 |
| 48 | 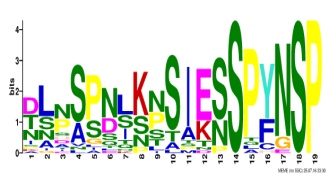 | [DT]L[NP][SA][PS][ND][LS][KS][NP]SI[EK][SN]SP[YF]NSP | 1.6e-149 |
| 49 | 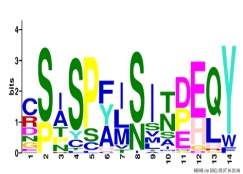 | [CR][SP][IA]SP[FAY][ILM][SN]I[TN][DPE][EH][QL]Y | 7.1e-140 |
| 50 | 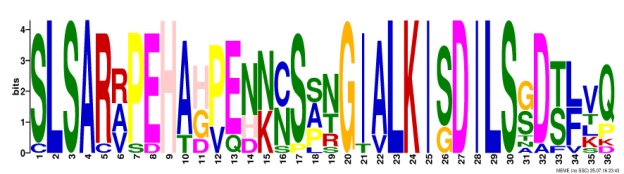 | SLSAR[RAV]PEHA[HG][PV]E[NH][NK][CN]S[ASP][NT]GIALKI[SG]DILS[GS]D[TS][LF]VQ | 1.2e-206 |
